# 

**Published:** 1923-08

**Authors:** 


### Contents

The Life of Pierre Fauchard (1678-1761) .......... 337

The Properties and Functions of Saliva .......... 357

The Organisms Found in the Periodontal Infections, and Their Relation to Toxaemia .......... 364

Indents .......... 378

